# A modern way to teach and practice manual therapy

**DOI:** 10.1186/s12998-024-00537-0

**Published:** 2024-05-21

**Authors:** Roger Kerry, Kenneth J. Young, David W. Evans, Edward Lee, Vasileios Georgopoulos, Adam Meakins, Chris McCarthy, Chad Cook, Colette Ridehalgh, Steven Vogel, Amanda Banton, Cecilia Bergström, Anna Maria Mazzieri, Firas Mourad, Nathan Hutting

**Affiliations:** 1 School of Health Sciences, Queens Medical Centre, University of Nottingham, Nottingham, NG7 2HA UK; 2 Allied Health Research Unit, University of Central Lancashire, Preston, PR1 2HE UK; 3 Centre of Precision Rehabilitation for Spinal Pain, School of Sport, Exercise and Rehabilitation Sciences, University of Birmingham, Edgbaston, Birmingham, B15 2TT UK; 4 Nottingham CityCare Partnership, Bennerley Rd, Nottingham, NG6 8WR UK; 5 School of Medicine, University of Nottingham, Queens Medical Centre, Nottingham, NG7 2HA UK; 6 Department of Orthopaedics, West Herts Hospitals Trust, Watford, WD18 0HB UK; 7 School of Physiotherapy, Manchester Metropolitan University, Manchester, M15 6GX UK; 8 Department of Orthopaedics, Duke University, 200 Morris Street, Durham, NC 27701 USA; 9 School of Sport and Health Sciences, University of Brighton, Darley Rd, Eastbourne, BN20 7UR UK; 10 Clinical Neuroscience, Trafford Building, Brighton and Sussex Medical School, University of Sussex, Brighton, BN1 9PX UK; 11 University College of Osteopathy, 275 Borough High St, London, SE1 1JE UK; 12 Department of Clinical Sciences, Obstetrics and Gynecology, Umeå University, S-90187 Umeå, Sweden; 13 The School of Soft Tissue Therapy, Exmouth, Devon EX8 1DQ UK; 14 Department of health, LUNEX, Differdange, 4671 Luxembourg; 15 Luxembourg Health & Sport Sciences Research Institute A.s.b.l., 50, Avenue du Parc des Sports, Differdange, 4671 Luxembourg; 16 Department of Occupation and Health, School of Organization and Development, HAN University of Applied Sciences, Nijmegen, the Netherlands

**Keywords:** Manual Therapy, Evidence-based healthcare, Person-centred healthcare, Physiotherapy, Osteopathy, Chiropractic, Soft-tissue therapy

## Abstract

**Background:**

Musculoskeletal conditions are the leading contributor to global disability and health burden. Manual therapy (MT) interventions are commonly recommended in clinical guidelines and used in the management of musculoskeletal conditions. Traditional systems of manual therapy (TMT), including physiotherapy, osteopathy, chiropractic, and soft tissue therapy have been built on principles such as *clinician-centred assessment*, *patho-anatomical reasoning,* and *technique specificity.* These historical principles are not supported by current evidence. However, data from clinical trials support the clinical and cost effectiveness of manual therapy as an intervention for musculoskeletal conditions, when used as part of a package of care.

**Purpose:**

The purpose of this paper is to propose a modern evidence-guided framework for the teaching and practice of MT which avoids reference to and reliance on the outdated principles of TMT. This framework is based on three fundamental humanistic dimensions common in all aspects of healthcare: *safety*, *comfort*, and *efficiency*. These practical elements are contextualised by positive *communication*, a collaborative *context*, and *person-centred care*. The framework facilitates best-practice, reasoning, and communication and is exemplified here with two case studies.

**Methods:**

A literature review stimulated by a new method of teaching manual therapy, reflecting contemporary evidence, being trialled at a United Kingdom education institute. A group of experienced, internationally-based academics, clinicians, and researchers from across the spectrum of manual therapy was convened. Perspectives were elicited through reviews of contemporary literature and discussions in an iterative process. Public presentations were made to multidisciplinary groups and feedback was incorporated. Consensus was achieved through repeated discussion of relevant elements.

**Conclusions:**

Manual therapy interventions should include both passive and active, person-empowering interventions such as exercise, education, and lifestyle adaptations. These should be delivered in a contextualised healing environment with a well-developed person-practitioner therapeutic alliance. Teaching manual therapy should follow this model.

**Supplementary Information:**

The online version contains supplementary material available at 10.1186/s12998-024-00537-0.

## Background

Musculoskeletal (MSK) conditions are leading contributors to the burden of global disability and healthcare [1]. Amongst other interventions, manual therapy (MT) has been recommended for the management of people with MSK conditions in multiple clinical guidelines, for example [2, 3].

MT has been described as the deliberate application of externally generated force upon body tissue, typically via the hands, with therapeutic intent [4]. It includes touch-based interventions such as thrust manipulation, joint mobilisation, soft-tissue mobilisation, and neurodynamic movements [5]. For people with MSK conditions, this therapeutic intent is usually to reduce pain and improve movement, thus facilitating a return to function and improved quality of life [6]. Patient perceptions of MT are, however, vague and sit among wider expectations of treatment including education, self-efficacy and the role of exercise, and prognosis [7].

Although the teaching and practice of MT has invariably changed over time, its foundations arguably remain unaltered and set in biomedical and outdated principles. This paper sets out to review contemporary literature and propose a revised model to inform the teaching and practice of MT.

The aim of this paper is to stimulate debate about the future teaching and practice of manual therapy through the proposal of an evidence-informed re-conceptualised model of manual therapy. The new model dismisses traditional elements of manual therapy which are not supported by research evidence. In place, the model offers a structure based on common humanistic principles of healthcare.

### Consenus methodology

**Fig. 1:**
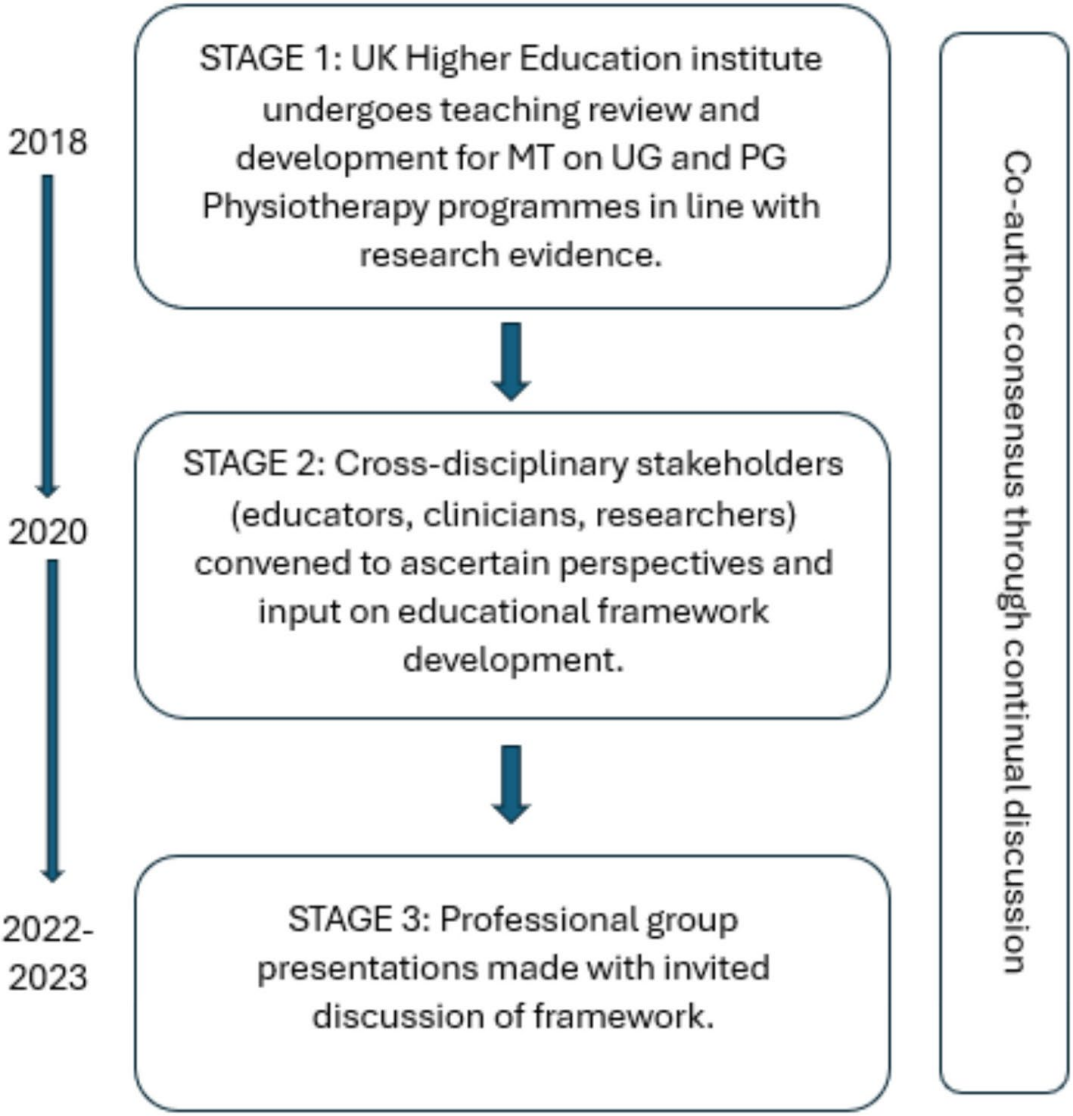
Summary and timeline of iterative consensus process for development of framework (MT: Manual Therapy; UG: Undergraduate; PG: Postgraduate)

We present the literature synthesis and proposed framework as a consensus document to motivate further professional discussion developed through a simple three-stage iterative process over a 5-year period. The consensus methodology was classed as educational development which did not require ethical approval. Stage 1: a change of teaching practice was adopted by some co-authors (VG, RK, EL) on undergraduate and postgraduate Physiotherapy programmes at a UK University in 2018. This was a result of standard institutional teaching practice development which includes consideration of evidence-informed teaching. Stage 2: Input from a broader spectrum of stakeholders was sought, so a group of experienced, internationally-based educators, clinicians, and researchers from across the spectrum of manual therapy was convened. Perspectives were elicited through discussions in an iterative process. Stage 3: Presentations were made by some of the co-authors (VG, RK, SV, KY) to multidisciplinary groups (UK, Europe, North America) and feedback via questions and discussions was incorporated into further co-author discussions on the development of the framework. Consensus was achieved through repeated discussion of relevant elements. Figure 1 summarises the consensus methodology.

### Clinical & cost effectiveness of manual therapy

Manual therapy has been suggested to be a valuable part of a multimodal approach to managing MSK pain and disability, for example [8]. The majority of recent systematic reviews of clinical trials report a beneficial effect of MT for a range of MSK conditions, with at least similar effect sizes to other recommended approaches, for example [9]. Some systematic reviews report inconclusive findings, for example [10], and a minority report effects that were no better than comparison or sham treatments, for example [11].

Potential benefits must always be weighed against potential harms, of course. Mild to moderate adverse events from MT (e.g. mild muscle soreness) are common and generally considered acceptable [12], whilst serious adverse events are very rare and their risk may be mitigated by good practice [13]. MT has been reported by people with MSK disorders as a preferential and effective treatment with accepted levels of post-treatment soreness [14].

MT is considered cost-effective [15] and the addition of MT to exercise packages has been shown to increase clinical and cost-effectiveness compared to exercise alone in several MSK conditions [16–23]. Further, manual therapy has been shown to be less costly and more beneficial than evidence-based advice to stay active [24].

In summary, MT is considered a useful evidence-based addition to care packages for people experiencing pain and disability associated with MSK conditions. As such, MT continues to be included in national and international clinical guidelines for a range of MSK conditions as part of multimodal care.

### Principles of traditional manual therapy (TMT)

Manual therapy has been used within healthcare for centuries [4] with many branches of MT having appeared (and disappeared) over time [25]. In developed nations today, MT is most commonly utilised by the formalised professional groups of physiotherapy, osteopathy, chiropractic, as well as groups such as soft tissue therapists. All of these groups have a history that borrows heavily from traditional healers and bone-setters [26].

Although there are many elements of MT, three principles appear to have become ubiquitous within what we shall now refer to as ‘traditional manual therapy’ (TMT): *clinician-centred assessment*, *patho-anatomical reasoning*, and *technique specificity* [27–30]. These principles continue to influence the teaching and practice of manual therapy over recent years, for example [31].

However, they have become increasingly difficult to defend given a growing volume of empirical evidence to the contrary.

### Traditional manual therapy (TMT) principles: origins and problems

#### Clinician-centred assessment

TMT has long had an emphasis on what we shall refer to as *clinician-centred assessments*. Within this, we claim, is an assumption that clinical information is both highly accurate and diagnostically important, for example [32]. Clinician-centred assessments include, for example, routine imaging, the search for patho-anatomical 'lesions’ and asymmetries, and specialised palpation. Although the focus of this paper is on the ‘hands-on’ examples of client-centred assessment, the notion of imaging is presented below to expose some of the flaws in the underlying belief system for TMT.

The emphasis on clinician-centred assessments has probably been driven, in part, by a desire for objective diagnostic tests which align well with gold-standard imaging. Indeed, since the discovery of x-rays, radiological imaging been used as an assessment for spinal pain – and a justification for using spinal manipulation – particularly in the chiropractic profession [33]. Contrary to many TMT claims, X-ray imaging is not without risk [34]. Additionally, until relatively recently (with the advent of magnetic resonance imaging) it was not widely appreciated that patho-anatomical ‘lesions’ believed to explain MSK pain conditions were nearly as common in pain-free individuals as those with pain [35]. Accordingly, the rates of unnecessary treatments, including surgery, are known to increase when imaging is used routinely [36]. For patients with non-specific low back pain, for example, imaging does not improve outcomes and risks overdiagnosis and overtreatment [37]. Hence, despite being objective in nature, the value of imaging for many MSK pain conditions (particularly spinal pain) has reduced drastically with clinical guidelines across the globe recommending against routine imaging for MSK pain of non-traumatic origin [38]. Even so, the practice of routine imaging continues [39].

Hands-on interventions are inextricably related to hands-on assessment [40], and often associated with claims of ‘specialisation’ [41]. By this we mean where a great level of training and precision are claimed to be necessary for influencing the interpretation of assessment findings, treatment decisions, and/or treatment outcomes. Implicit within this claim is that therapists who are unable to achieve such precision are not able to perform MT to an acceptable level (and thereby are not able to provide benefit to patients).

There are numerous studies that cast doubt over claims of highly specialised palpation skills. Palpation of anatomical landmarks does not reach a clinically acceptable level of validity [42]. Specialised motion palpation does not appear to be a good method for differentiating people with or without low back pain [43]. Poor content validity of specialised motion tests have been reported, in line with a lack of acceptable reference standards [44]. Palpable sensations reported by therapists are unlikely to be due to tissue deformation [45]. Furthermore, the delivery of interventions based on specialised palpatory findings is no better than non-specialised palpation [46]. Generally poor reliability of motion palpation skills has been reported, for example [47] and appear to be independent of clinician experience or training, for example [48]. Notably, *person-centred* palpation—for pain and tenderness for example—has slightly higher reliability, but is still fair at best [49].

This does not mean that palpation is of no use at all though; just that effective manual therapy does not depend upon it. For example, expert therapists can display high levels of interrater reliability during specialised motion palpation [50]. Focused training can improve the interrater reliability of specialised skills [51]. However, the validity of the phenomenon remains poor. Given the weight of the evidence and consistency of data over recent decades, we suggest that the role of clinician-centred hands-on assessment is no longer central to contemporary manual therapy.

### Patho-anatomical reasoning

The justification for selecting particular MT interventions has historically been based upon the patho-anatomical status of local peripheral tissue [52–55]. Patho-anatomical reasoning, we propose, is the framework that links clinician-centred assessments to the desire for highly specific delivery of MT interventionsKey to this is the relationship between a patho-anatomic diagnosis and the assumed mechanisms of action of the intervention employed.

Theories for the mechanisms of action of MT interventions are many. Some of the most prominent include reductions of disc herniations [56], re-positioning of a bone or joint [32], removal of intra-articular adhesions [57], changes in the biomechanical properties of soft tissues [58], central pain modulation [59], and biochemical changes [60]. These theories have been used to justify the choice of certain interventions: a matching of diagnosis (i.e., existence of a lesion) to the effect of treatment takes place. However, most of these mechanistic theories either lack evidence or have been directly contested [61].

The causal relationship between proposed tissue-based factors such as posture, ergonomic settings, etc. and painful experience has also been disputed [62]. Although local tissue stiffness has been observed in people with pain, this is typically associated with neuromuscular responses, rather than patho-anatomical changes at local tissue level [63–66]. Overall, although some local tissue adaptions have been identified in people with recurrent MSK pain, this is inconsistent and the evidence is currently of low quality [67] are generally limited to short-term follow-up measures [68].

### Technique specificity

TMT techniques have been taught with an emphasis that a particular direction, ‘grade’ of joint movement, or deformation of tissue at a very specific location in a certain way, is required to achieve a successful treatment outcome.

One problem with a demand for technique specificity in manual therapy is that an intervention does not always result in the intended effect. For example, posteroanterior forces applied during spinal mobilization consistently induce sagittal rotation, as opposed to the assumed posteroanterior translation, for example [69]. Furthermore, irrespective of the MT intervention chosen, restricting movements to a particular spinal segment is difficult and a regional, non-specific motion is typically induced, for example [70].

To support technique specificity, comparative data must repeatedly and reproducibly show superiority of outcome from specific MT interventions over non-specific MT, which is consistently not observed [71–73]. Some studies have demonstrated localised effects of targeted interventions [74] but there appears to be no difference in outcome related to: the way in which techniques are delivered [75]; whether technique selection is random or clinician-selected [41]; or variations in the direction of force or targeted spinal level [76]. Conversely, there *is* evidence that non-specific technique application may improve outcomes [77–79]. Further, sham techniques produce comparable results to specialised approaches [11].

Passive movement and localised touch have been associated with significant analgesic responses [80]. These data indicate the presence of an analgesic mechanism. Unfortunately, mechanistic explanation for the therapeutic effects of MT upon pain and disability still remain largely in a ‘black box’ state [81]. Nevertheless, there are several plausible mechanisms of action to explain the analgesic action of MT interventions, including the activation of modulatory spinal and supraspinal responses [82–85]. In support of this, MT interventions have been associated with a variety of neurophysiological responses [61]. However, it must be acknowledged that these studies provide mechanistic evidence based on association, which is insufficient to make causal claims [86]. Importantly, none of these neurophysiological responses have been directly related to either the analgesic mechanisms or clinical outcome and may therefore be incidental.

There is evidence that MT does not provide analgesia in injured tissues [87, 88]. Conversely, MT has been shown to decrease inflammatory biomarkers [89–93], although these changes have not been evaluated in the longer-term, nor associated with clinical outcomes.

### A modern framework for manual therapy

We propose a new direction for the future of MT in which the teaching and practice of this core dimension of MSK care are no longer based on the traditional principles of *clinician-centred assessment*, *patho-anatomical reasoning*, and *technique specificity*.

In doing so, this framework places MT more explicitly as part of person-centred care and appeals to common principles of healthcare, best available evidence, and contemporary theory which avoids unnecessary and over-complicated explanations of observed effects. The framework is simple in terms of implementation and delivery and contextualised by common elements of best practice for healthcare, in line with regulated standard of practice, e.g., [94–97]. Our proposal simply illustrates the operationalisation of these common elements through manual therapy.

Too much emphasis has been given to clinician-centred assessments and this should be rebalanced with an increased use of patient-centred assessments, such as a thorough case history, the use of validated patient-reported outcome measures (PROMS), and real-time patient feedback during assessments.

The new framework considers fundamental and humanistic dimensions of touch-based therapies, such as non-specific neuromodulation, communication and sense-making, physical education, and contextual clinical effectiveness. This aligns to contemporary ideas regarding therapeutic alliance and a move towards genuinely holistic healthcare [98, 99]. The framework needs to be “open” in order to represent and allow expression of the complexity of the therapeutic encounter. However, to prevent the exploitation of this openness the framework is underpinned by evidence, and any manual therapy approaches without plausible and measurable mechanisms are not supported.

**Fig. 2:**
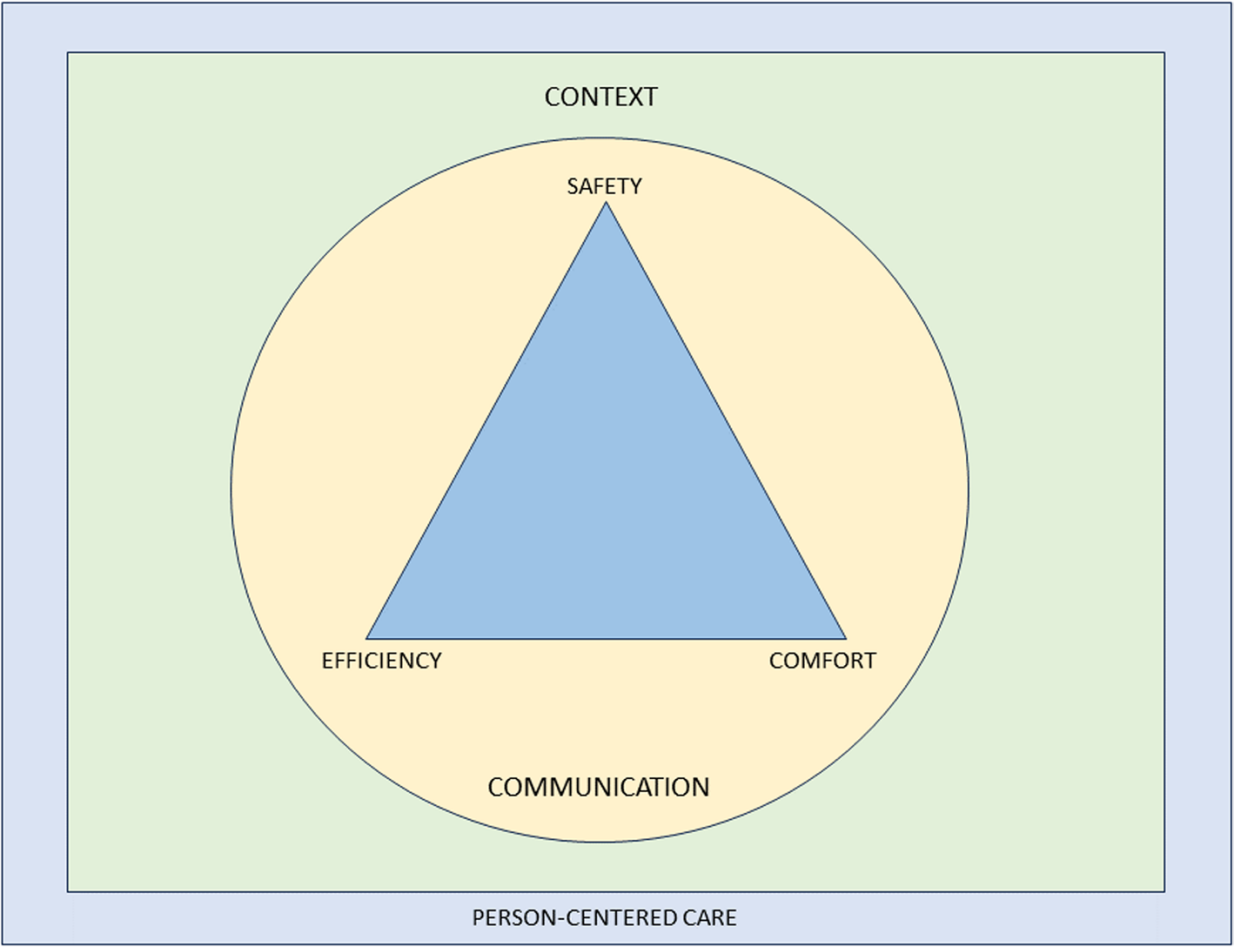
Representation of a modern teaching and practice framework for manual therapy. The image is purposefully designed to be simple, and has been developed primarily to be used as a teaching aid. When displayed in a learning environment, learners and clinicians can quickly refer to the image to check their practice against each element. To keep the image clear, each element of the image is described in detail in the text below”

To provide the best care, common healthcare elements such as the safety and comfort of the person seeking help and therapist must be considered, and care should be provided as efficiently as possible. Our framework embraces these dimensions and employs an integration of current evidence. It is transdisciplinary in nature and may be adopted by all MT professions. Figure 1 provides a graphical representation of the framework. It is acknowledged that all components overlap, relate, and influence each. There are two main components: the practical elements on the inside, comprised of safety, comfort, and efficiency, and the conceptual themes on the outer regions, consisting of communication, context, and person-centred care Fig. 2.

### Practical elements

#### Safety

Safety for people seeking help is a primary concern for all healthcare providers, with the aims to “*prevent and reduce risks, errors and harm that occur to patients [sic] during provision of health care… and to deliver quality essential health services*” [100]. This, and the notion of safety more generally (including that of the therapist), should be central to way MT is taught and practised.

A fundamentally safe context should be created where there is an absence of any obvious danger or risk of harm to physical or mental health. Consideration should be given to ensuring that communication and consent processes are orientated towards the safety of both the person seeking help and the therapist. The therapist should pay attention to any sense of threat that could be present in the physical, emotional, cognitive and environmental domains of the clinical encounter, and use skilful communication to mitigate anxiety about the assessment or therapeutic process.

Safety should also be considered in the clinical context of the assessment and treatment approach, ensuring that relevant and meaningful safety screenings have been undertaken [67, 101]. There remains a need for good, skilful practice and development of manually applied techniques, but this can be achieved without reference to the principles of TMT and without the dogma of a proprietary therapeutic approach.

#### Comfort

*Comfort* suggests that both the person seeking help and the therapist are physically and emotionally content during the assessment and therapeutic process. For example, the person seeking help is agreeable with any necessary state of dress (sociocultural difference should be considered); the person is relaxed and untroubled in whatever position they are in, and is adequately supported whether sitting, standing or recumbent during assessment and treatment; the therapist is comfortable with their positioning and posture; any discomfort produced by the therapeutic process is negotiated and agreed. Any physical mobilisation or touch should be applied with respect to the feedback from the person in relation to their comfort, rather than a pre-determined force based on the notion of resistance. This process requires clinical phronesis, sensitivity, responsivity, dexterity, and embodied communication [102].

#### Efficiency

The therapeutic process should be undertaken in a well-organised, competent manner aiming to achieve maximum therapeutic benefit with minimum waste of effort, time, or expense. To enhance the efficiency dimension, the assessment and therapeutic process should be an integral part of a holistic educational and/or activity-based approach to the management of the people which might also address psychological, nutritional, or ergonomic aspects of care, while being aware of social determinants to health. Recommendations exist which serve as a useful guide for enhancing care and promoting self-management in an efficient way [103].

A principle of this new model of MT is that therapists should not lose sight of the goals they develop with the people they help and ensure that there is coherence between their management aims and their techniques. Therapists should aim to support a person’s self-efficacy and use active approaches to empower them in their recovery. The overall number of therapeutic applications should be made in the context of fostering therapeutic alliance and supporting people to make sense of their situation and symptoms. This should be informed by contemporary views of the effects of manual therapy, emphasising a “physical education process” to promote sense-making and self-efficacy in alliance with the people they aim to help.

Clinical interactions need to be reproducible under a person’s own volition, serving to enhance self-empowerment. For example, someone could be taught how to “self-mobilise” if a positive effect is found with a particular therapeutic application. This should be appropriately scaffolded with behavioural change principles and functional contextualism that promote autonomy and self-management, rather than inappropriate reliance on the therapist [103, 104].

An important and emergent notion from the proposed model is to question what constitutes *indications* for MT given that the model excludes traditional factors which would have informed whether manual therapy is indicated or not for a particular person. The response to this sits within the efficiency and safety dimensions: MT can be beneficial as part of a multi-dimensional approach to management across a broad population of people with musculoskeletal dysfunction, with no evidence to suggest any clinician-centered or patho-anatomical finding influences outcomes. The choice of whether or not to include MT as part of a management strategy should therefore be a product of a lack of contraindications and shared-decision making.

This framework aligns with evidence-based propositions that effectiveness and efficiency in assessment, diagnosis, and outcomes are not reliant on the therapist’s skill set of specialised elements of TMT, but rather other factors—for example variations in pain phenotypes [5].

### Conceptual themes

#### Communication

*Communication* is the overriding critical dimension to the whole therapeutic process and should be aimed at addressing peoples’ fundamental needs to make sense of their symptoms and path to recovery. The delivery and uptake of the therapy should therefore be operationalised in a communication process that meaningfully represents shared-decision making and the best possible attempt to contextualise the therapy in positive and evidence-informed explanations of the process and desired effects [105].

Within a therapeutic encounter, practitioners must give the time to listen to peoples’ accounts and explanations of their symptoms, including their ideas about their cause [106]. The assessment and diagnostic process should be a shared endeavour, for example, the negotiation of symptom reproduction. This should be done in a manner that facilitates sense-making, and which simultaneously encourages people to move on from unhelpful beliefs about their symptoms [107, 108], encouraging understanding of the uncertain nature of pain and injury. Person-centered communication requires attention to *what* we communicate and *how* we communicate across the entire clinical interaction including interview, examination, and management planning [109]. Therapists need to be open, reflective, aware and responsive to verbal and non-verbal cues, and demonstrate a balance between engaging with people (e.g. eye-gaze) and writing/typing notes during the interview [110–112].

People should be given the opportunity to discuss their understanding of the diagnosis and options for treatment and rehabilitation. The decision-making process is dialogical, in which alternative options to the offered therapy should also be discussed with the comparative risks and benefits of all available management options, including doing nothing [113, 114].

The therapist must fully appreciate the potential consequences of touch without consent. Continual dialogue should ensure that all parties are moving towards mutually agreed goals. The context of the therapy should be explicitly communicated to give appropriate context for any particular intervention as part of a holistic, evidence-based approach [115–117]. Therapists should be aware that their own beliefs can affect the way they communicate with their people; in the same way, a person’s context affects how they communicate what they expect from their treatment [107, 118–120]. The construction of contextual healing scenarios which support positive outcomes, whilst minimising nocebic effects, is critical to effective healthcare [121–123].

There is a growing academic interest in the nature, role, and purpose of social and affective touch, and any re-framing of MT should consider touch as a means of communication to develop and enhance cooperative communications and strengthen the therapeutic relationship [124–129]. It can be soothing for a person in pain to experience the caring touch of a professional therapist [130]; on the other hand, probing, diagnostic, and touch can be experienced as alienating [131–133]. Touch can alter a person’s sense of body ownership and their ability to recognise and process their emotions by modulating interoceptive precision [129, 134, 135], and intentional touch may be perceived differently from casual, unfocussed touch [136, 137]. There is also a thesis that touch generates shared understanding and meaning [138–140]. This wider appreciation of touch should be embedded in modern MT communication.

#### Context

The contextual quality of a person’s experience of the therapeutic encounter can affect satisfaction and clinical outcomes [141–145]. The context in which therapeutic care takes place should therefore be developed to enhance this experience. There could be very local, practical aspects of the context, such as the type of passive information available in the clinical space, e.g. replacing biomedical and pathological imagery and objects with positive, active artefacts; judicious and thoughtful organisation and use of treatment tables to discourage a sense of passivity and disempowerment; allocating a comfortable space where communication can take place; colour schemes and light sources which facilitate positivity; ensuring consistency through all clinical and administrative staff promoting encouraging and non-nocebic messages. Importantly, the way the therapist dresses influences peoples’ perception of their healthcare experience [146, 147], and that in turn should be contextually and culturally sensitive [148–150].

Beyond the local clinical space is the broader social environment. The undertaking of MT should serve a role in a person’s engagement with their social environment. For example, someone returning home after engaging with their therapist and disseminating positive health messages within their home and social networks; people acting as advocates for self-empowered healthcare. Furthermore, early data have demonstrated that aligning treatment with the beliefs and values of culturally and linguistically diverse communities enhances peoples’ engagement with their healthcare [151].

### Person-centred care

Here we borrow directly from one of the most established and clinically useful definitions of Person-Centered Medicine [152]:

*“(Person-Centered Medicine is) an affordable biomedical and technological advance to be delivered to patients* [sic] *within a humanistic framework of care that recognises the importance of applying science in a manner that respects the patients* [sic] *as a whole person and takes full account of [their] values, preferences, aspirations, stories, cultural context, fears, worries and hopes and thus that recognises and responds to [their] emotional, social and spiritual necessities in addition to [their] physical needs” *[152]*, p219.*

Person-centred care incorporates a person’s perspective as part of the therapeutic process. In practice, therapists need to communicate in a manner that creates adequate conversational space to elicit a person’s agenda (i.e. understanding, impact of pain, concerns, needs, and goals), which guides clinical interactions. This approach encourages greater partnership in management [109, 153, 154].

A roadmap outlining key actions to implement person-centeredness in clinical practice has been outlined in detail elsewhere [155]. This includes screening for serious pathology, health co-morbidities and psychosocial factors; adopting effective communication; providing positive health education; coaching and supporting people towards active self-management; and facilitating and managing co-care (when needed) [154].

It is critical and necessary now to make these features explicit and central to the revised model of MT proposed in this paper. We wish to identify common ground across all MT professions in order to achieve a trans-disciplinary understanding of the evidence supporting the use of MT.

We acknowledge that our arguments here are rooted in empiricism and deliberately based on available research data from within the health science disciplines. We also acknowledge that there is a wider debate about future directions in person-centred care arising from the current evolution of the evidence-based health care movement, which has pointed to the need to learn more about peoples’ lived experiences, to redefine the model of the therapeutic relationship. Although beyond the scope of this paper, a full exploration of modern health care provision involves reconsideration of the ethics and legal requirements of communication and shared decision-making [156–159]. The authors envision this paper as a stimulus for self-reflection, stakeholder discussions, and ultimately change that can positively impact outcomes for people who seek manual therapy interventions.

## Conclusions

Manual therapy has long been part of MSK healthcare and, given that is likely to continue. Current evidence suggests that effectiveness does not rely on the traditional principles historically developed in any of the major manual therapies. Therefore, the continued teaching and practice based on the principles of *clinician-centred palpation*, *patho-anatomical reasoning*, and *technique specificity* are no longer justified and may well even limit the value of MT.

A revised and reconceptualised framework of MT, based on the humanistic domains of safety, comfort and efficiency and underpinned by the dimensions of communication, context and person-centred care will ensure an empowering, biopsychosocial, evidence-informed approach to MSK care. We propose that the future teaching and practice of MT in physiotherapy, osteopathy, chiropractic, and all associated hands-on professions working within the healthcare field should be based on this new framework.

### Supplementary Information

**Supplementary Material 1.**

## Data Availability

N/A.
