# Supplementary material for: A modern way to teach and practice manual therapy
Source: Chiropr Man Therap. 2024 May 21;32:17. doi: 10.1186/s12998-024-00537-0 (PMC11110311; doi:10.1186/s12998-024-00537-0)
Supplement: Supplementary file 1 — Supplementary Material 1. [file 12998_2024_537_MOESM1_ESM.docx]

# CASE STUDY 1 – TEACHING & LEARNING

*Scenario*: Year 1 Undergraduate Manual Therapy class. 50 students, 2 educators

*Task*: Learning how to mobilise the cervical spine for assessment and therapeutic purposes.

*Process*: Educators demonstrate on student model the basics of setting up a safe treatment table and floor space and facilitating (verbally or physically as necessary) the model onto the treatment table. The educator reviews the indications, contraindications, and non-indications for manual therapy addressed in previous modules and works with the model to establish a comfortable position, in which efficient MT can be undertaken. The educators demonstrate passive movements of the head and neck, and reviews relevant elements of contextual-focussed therapeutic communication as well as the influence of the context and contextual factors, covered in separate modules.

*Interaction*: The students now practice this technique. Some students ask where they should put their hands, and how they should hold the models. The educators respond with asking both the student and their model what feels most comfortable to both of them, bearing in mind the movement required. Students then work with each other to achieve comfortable handling.

A student then asks if there could be any harm for the model. The educator then asks the student “how do you think you could harm someone doing this?” and the student reflects on their understanding of local functional anatomy of neurovascular structures around the neck. The student and model then discuss where they wouldn’t put their hands.

Another student asks which direction of movement they should be doing, how far should they move the head and neck, and how many times. The educator says as this is a practice session with healthy models, they should practice as much as possible, in all directions in the allocated time, but stop if either they or the model becomes uncomfortable. They then ask “how far and often would you do this is practice”. The educator responds by beginning a conversation about clinical reasoning, and establishing the severity, irritability, nature and therapeutic goals of the person’s complaint, as covered in earlier modules. To enhance efficiency, the students are encouraged to work with the models to role-play movement preferences, nature of mobilisation (sustained; oscillatory, etc.); consider severity, irritability for force and comfort, etc. The conversation leads to the students learning about how treatment is influenced by a person’s individual status, and that the treatment should be for the shortest possible duration in order to facilitate active, functional, goal-related tasks.

After working out a comfortable method of executing a posterior-anterior accessory movement in the cervical spine, a student asks how hard they should push. The educator initiates a similar conversation about reasoning, safety, comfort, efficiency, and goal-focussed treatment.

The session is halted for a few minutes whilst the educators again remind the students about how they might best be communicating with the patient to ensure an optimal contextual healing scenario. They are also reminded of the possible neuromodulatory and cortical responses they are trying to harness.

*Analysis*: students have learnt a process of handling which is safe, comfortable, and efficient whilst considering their communication and context. They relate their learning to previous and parallel learning on person-centred care, The key thing is that the tutor continually draws out the relationships to other holistic and biopsychosocial learning modules. There has been no reference to or guidance from traditional principles like palpating for specific movement changes or dysfunctions, specific rule-based direction and force preferences, nor encouragement that local tissue responses and changes should form part of the reasoning process. Skill is being developed, but the student has avoided pedagogical scaffolding from outdated principles of traditional manual therapy.

# CASE STUDY 2 – CLINICAL PRACTICE

*Scenario*: David is 54-year-old male with 2 years of neck pain and headaches. Limited movement into right rotation and flexion, due to pain and stiffness.

*Task*: Therapist aims to work with David to help him return to better and more comfortable work as an electrician.

*Process*: The therapeutic relationship is developed by careful and active listening during conversations about lifestyle, David’s understandings and beliefs about his painful experience, expectations and goals, and exercise. David has frequent bouts off work because of the pain, and so is increasingly financially struggling and considering bankruptcy. He is otherwise fit and well and active, and his only respite for self-care and reflection is recreational cycling, whereby he covers around 100 miles a week.

*Interaction*: Goal-setting is established with David and he mind-maps the variables he thinks are contributing to his pain. He has tried doing neck exercises, general body exercise and neural-sensitivity exercise as given by previous therapists. He has had previous treatment sessions involving manual therapy, for which he has had little or short-lived responses. David has some anxiety and fear about moving his head into flexion and rotation and is apprehensive when asked to do this. A conversation is developed whereby the therapist and David agree that a few minutes of the therapist moving David’s head and neck might help David’s confidence in actively moving. During this conversation David reveals his beliefs about previous MT sessions and that he thought previously therapists were trying to ‘stretch his joints’ and ‘release muscle tension’. One therapist had performed high-velocity thrust manipulations with the intention of ‘forcing a vertebra back in’. The therapist explains that there are more contemporary explanations of the effects of MT whilst being mindful of how this might relate to his existing beliefs. The therapist proposes that if David would like, they can work together to see if passive movement can help reassure that movement is OK and perhaps reduce some temporary discomfort. A materially relevant informed consent process is successfully completed.

The therapist works with David to establish a safe and comfortable position, and David chooses to lie on his back. The therapist asks David what is the most comfortable and reassuring way to hold his neck, and then asks David which movement they should do first. David says rotation, and the therapist asks him to visualise his neck and head movement, knowing that each time his head is moved, his thoughts and beliefs about movement have the possibility to change, especially if he is feeling comfortable during the movement. After 2 or 3 minutes of gentle repeated movement into a variety of rotation and flexion, David sits up for a couple of minutes and then volunteers to move by himself. He is happy that he can move into rotation and flexion without the previous anxiety. The therapist reminds David that although local movement has obviously occurred, there has been nothing structurally ‘corrective’ about what they have done and that he is most likely experiencing some transient analgesia and self-reassurance. This is enough however to make David a little more confident in movement. A plan of goal-focussed movement is then co-produced to help maintain and then develop further David’s confidence and reassurance in moving.

*Analysis*: MT has been undertaken, but with no reference to specific palpation findings, specific joint force or direction rules, or reinforcement of patho-anatomical beliefs. Three minutes of safe, comfortable and efficient passive movement has possibly influenced neurological phenomena and produced some cortical changes. David feels empowered to re-evaluate his beliefs about his pain and has developed his own strategies for painful relapses during work. Communication has been direct and person-centred, and contextual healing has been established.
